# Solution Characterization and Initial Crystallization Studies of TrbB from the F Plasmi

**DOI:** 10.1063/4.0001099

**Published:** 2025-10-27

**Authors:** Maya Soko, Gerald Audette

**Affiliations:** York University, Toronto, Ontario, Canada

## Abstract

Secretion systems, found across gram-negative and gram-positive bacteria, are large multi-protein complexes responsible for the transport of genetic material and proteins across phospholipid membranes, with dedicated systems in place for the secretion of virulence factors into host cells or the environment. One such pathway can be found in Gram-negative bacteria bearing conjugative F-like plasmids is known as the Type IV Secretion System (T4SSF). This system comprises multiple proteins responsible for cell proliferation, transport of virulence factors and antibiotic resistance genes through bacterial conjugation. Bacterial conjugation involves the assembly of a transmembrane conjugative pili for Gram negative bacteria bearing F-like plasmids, that is responsible for plasmid DNA transfer. TrbB is a periplasmic protein encoded by the F- plasmid transfer region (tra), with disulfide isomerase and reductase activity. TrbB is proposed to be involved in the assembly of the F-pilus and aid in protein folding for other T4SSF proteins. Previous studies of full-length TrbB indicated a dynamic N-terminal domain likely responsible for protein-protein interactions. In the current study, we present recent SEC-MALS-SAXS data and initial high-throughput crystallization trials of an N- terminally truncated TrbB construct (TrbB37-161). SAXS data indicates several species in solution (monomer, dimer, tetramer), and crystallization trials identify several promising leads for further optimization. Noting the important role of conjugation in the the spread of antibiotic resistance genes and TrbB’s role in assembly of the conjugative F-pilus, further structural investigation may lead to improved strategies for curtailing the spread of antibiotic resistant pathogens with multidrug resistance

